# Recurrence and complications after laparoscopic inguinal hernia repair using a self-adherent mesh: a patient-reported follow-up study

**DOI:** 10.1007/s00464-025-11614-7

**Published:** 2025-02-24

**Authors:** Helle Lund, Lene Spanager, Azalie Caroline Riberholdt Winther, Mathias Gierløff, Katharina Sunekær, Jakob Kleif, Claus Anders Bertelsen

**Affiliations:** 1 Department of Surgery, Copenhagen University Hospital – North Zealand, Dyrehavevej 29, 3400 Hillerød, Denmark; 2 Department of Clinical Medicine, Faculty of Health and Medical Sciences, University of Copenhagen, Copenhagen, Denmark

**Keywords:** TAPP, Recurrence, Pain, Survey, Self-adherent mesh, Fixation

## Abstract

**Background:**

Recurrence and postoperative pain are major concerns after laparoscopic surgery for inguinal hernia. Follow-up on all patients is difficult and time consuming for both the hospital and the patient. We conducted a patient-reported follow-up study to estimate the rate of recurrence and postoperative pain in our department.

**Method:**

Patients undergoing the TAPP (TransAbdominal PrePeritoneal) procedure with a self-adherent mesh at Copenhagen University Hospital – North Zealand from 2016 to 2019 received an online survey about signs of recurrence, postoperative pain, and complications. Patients reporting signs of recurrence or pain were contacted and invited for a clinical examination if relevant. Forty-five randomly selected patients who did not report any symptoms of recurrence or pain were contacted by phone for validation.

**Results:**

871 patients received a questionnaire, and 546 responded, leaving a response rate of 62.7%. Median follow-up time was 34 months (IQR 23–47). The self-reported recurrence rate was 8.1% (95% CI: 6.0–11.0%). On examination, recurrence was diagnosed in 2.4% (95% CI: 1.4–4.1%) of the patients. When including the patients with self-reported recurrence who did not accept the offer of clinical examination, the recurrence rate was 3.8% (95% CI: 2.5–5.8%). Four patients (0.7%, 95% CI: 0.2–2.0%) underwent herniotomy for recurrence. The rate of chronic postoperative pain impairing daily activity was 0.5%.

**Conclusion:**

We found an acceptable low rate of recurrence and postoperative pain compared to other studies. The patient-reported recurrence rate was significantly higher than the clinical recurrence rate after the examination, indicating that patient-reported recurrence seems to overestimate true recurrence after TAPP.

## Background

Recurrence and persisting pain are considered some of the most important patient-related outcomes after inguinal hernia repair. Several large-scale studies have compared laparoscopic with open inguinal hernia repair, reporting no difference in recurrence rates but significantly lower early and persistent pain rates after laparoscopic repair [1–4].

Several studies have examined whether the fixation method (tackers, glue, staples, or self-gripping meshes) affects recurrence rates and pain after the TAPP (TransAbdominal PrePeritoneal) procedure and found no differences [5–7]. A meta-analysis of more than 1700 patients found that patients experienced less pain when the mesh was not fixated compared with fixation [8], and studies have not reported a higher recurrence rate with the non-fixating method [9–11].

Chronic postoperative pain has been defined as pain impacting daily activities lasting more than 3 months postoperatively [12]. The etiology is diverse, being neuropathic and non-neuropathic. Several risk factors have been identified for developing chronic pain, e.g., young age, female gender, history of chronic pain, high-intensity preoperative pain, and severe early postoperative pain [13–15]. Various studies have reported a 10–30% prevalence of persisting pain following hernia repair, depending on the criteria used [16, 17]. The HerniaSurge group states that the prevalence of persisting pain is 10–12%, hereof affecting daily activities in 0.5–6% [18].

For over a decade, the laparoscopic transabdominal preperitoneal (TAPP) procedure has been the primary modality for primary inguinal hernia repair for non-scrotal hernias at the Department of Surgery, Copenhagen University Hospital—North Zealand (NOH) in Hillerød Denmark. Since 2016, we have used a self-adherent mesh (Cousin 4D1216®). Initially, we fixated the mesh to Cooper’s ligament with 2 Endo Universal™ staples. As we observed the self-adherent properties of the mesh, we omitted the fixation.

This study assessed the self-reported recurrence and complication (pain, seroma, and hematoma) rates after laparoscopic inguinal hernia repair using the self-adherent Cousin 4D1216® mesh with or without fixation.

## Method

The study was a retrospective cohort study of patients undergoing laparoscopic inguinal hernia repair with a self-adherent mesh with the TAPP procedure at NOH from 2016 to 2019. NOH is a public hospital providing tax-financed free health to 320,000 of the 1.9 million inhabitants of the Capital Region of Denmark. Information was retrieved through patients’ electronic healthcare records. These data were supplemented by an online electronic questionnaire. The Patient Safety Department at NOH and the Data Protection Agency of the Capital Region of Denmark approved the study according to Danish legislation. The study is reported according to the STROBE statement [19].

### Population

Adult patients (age ≥ 18 years) undergoing elective TAPP procedure for a primary unilateral/bilateral inguinal or femoral hernia as outpatient or inpatient surgery at NOH during the study period were included. Patients were excluded if unable to speak proficient Danish, in case of recurrent hernia, emergency surgery, or if hernia repair was not performed or supervised by a hernia specialist.

### Data and data sources

Patients’ electronic healthcare records were reviewed regarding data on demographics, comorbidities, intraoperative findings, mesh fixation or non-fixation, and subsequent surgery for recurrence performed at any public hospital in the Capital Region of Denmark or the Region of Zealand. Data were registered in a REDCap database [20].

### Intervention

As a standard, all patients received Dexamethasone 8 mg, Ibuprofen 400 mg, and Paracetamol 1 g orally as preoperative medication. Surgery was performed as laparoscopic inguinal hernia repair with the standard TAPP technique using a self-adherent mesh size 12 × 16.5 cm (4D1216® mesh from Cousin, Cousin Surgery, France). The mesh is lightweight, 75% resorbable, with a surface mass of 120 ± 10 g/m3 initially and 30 ± 10 g/m3 after ended resorption. It is made of polypropylene coated with L-lactic acid. In the initial phase of the study, the Endo Universal Hernia Stapler® (Covidien) was used to fixate the mesh to Cooper’s ligament with two staples and to close the peritoneal defect. In 2018, we gradually started suturing the peritoneum with a self-closing running suture and stopped fixating the mesh.

Patients were discharged routinely on the same day of surgery according to the guidelines of the Danish Society of Anesthesiology and Intensive Care Medicine [21]. If patients did not meet the discharge criteria for outpatient surgery or did not have an adult at home during the first night, the patients stayed in the hospital overnight. Patients were discharged with Paracetamol, Ibuprofen, and Morphine on demand. As a standard, all outpatients received a call from a nurse on the first postoperative day. For patients operated on Fridays, the call took place on the following Monday. There was no other standard follow-up of the patients.

### Measurements and patient follow-up

All patients received an electronic questionnaire from the secured electronic mail service (e-books) provided to all Danish residents. The questionnaire contained questions regarding symptoms of recurrence, chronic pain, and postoperative hematoma and seroma. Pain was reported by the patients using the pain NRS (numeric rating scale). Non-responders received a reminder after one month. The questionnaire was developed by the authors using clear and easy-understandable Danish. Patients who reported symptoms of recurrence or pain in the groin were contacted by phone to validate the questionnaire response. If we could not confirm the absence of a relapse over the phone, patients were offered a physical examination at the hospital. If a recurrence could not be clinically excluded, ultrasonography or computerized tomography (CT) assessment was performed. Furthermore, 45 randomly selected patients who did not report any symptoms of recurrence or pain were contacted by phone for validation.

### Outcomes

The primary outcome was recurrence, defined as hernia repair for recurrence or confirmation of recurrence at clinical examination, ultrasonography, or CT scan. Sensitivity analyses were performed as a worst-case scenario. These included unverified patient-reported recurrences that did not undergo clinical assessment due to unwillingness to attend.

Secondary outcomes were pain in the groin area in proximity to the operation lasting for more than three months, hematoma, and seroma in the groin/external genitals developed during the first postoperative week. Finally, we assessed the sensitivity and specificity of the self-reported data by phone interviews.

### Statistics

Demographic and baseline continuous data are presented as median and interquartile range, and categorical data are presented as numbers and frequencies. Binary outcomes were analyzed using logistic regression, and continuous outcomes were analyzed using linear regression. Point estimates and corresponding 95% confidence intervals are reported. Model assumptions were checked using residual diagnostics, and when assumptions were not met, appropriate non-parametric analyses were used. All available data were used. No imputations were performed. A two-tailed *p* value ≤ 0.05 was considered significant. Analyses were performed using R statistical software, V.4.3.1 [22].

## Results

**Fig. 1:**
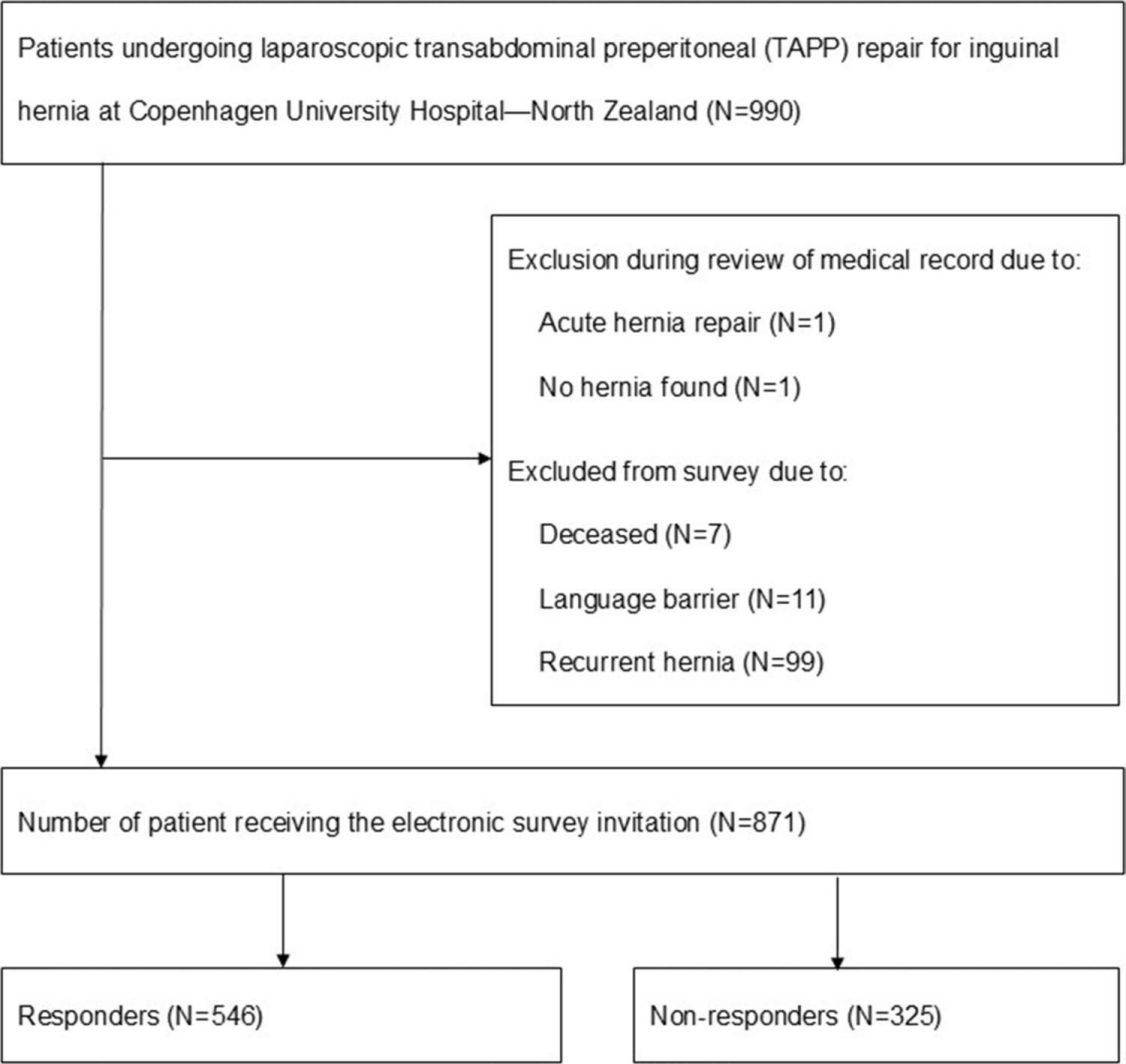
Inclusion and exclusion flow diagram

The questionnaire was sent to 871 patients, of whom 546 responded, resulting in a response rate of 62.7% (Fig. 1). Median follow-up time was 34 months (IQR 23–47). Patient demographics and intraoperative findings are presented in Table 1.

**Table 1:** Patient demographics and intraoperative findings stratified by survey responders and non-responders

	All patients *N* = 871	Non-responders *N* = 325	Responders *N* = 546	*p* value
Male	749 (86.0%)	275 (84,6)	474(86.8)	0.366
Median age (IQR) – years	64 (52–73)	57 (44–72)	66 (58–73)	< 0.001
ASA score – no. (%)				0.123
ASA I	363 (41.7%)	146 (44.9%)	217 (39.7%)	
ASA II	422 (48.5%)	143 (44.0%)	279 (51.1%)	
ASA III–IV	86 (9.9%)	36 (11.1%)	50 (9.2%)	
Median time from hernia repair to survey response (IQR)—months	33.7 (23.2–46.6)	32.4 (21.1–49.7)	33.7 (23.3–46.5)	0.997
Site of hernia—no. (%)				0.456
Right	429 (49.3%)	156 (48.0%)	273 (50.1%)	
Left	286 (32.9%)	115 (35.4%)	171 (31.4%)	
Bilateral	155 (17.8%)	54 (16.6%)	101 (18.5%)	
Fixation of mesh—no. (%)				0.164
No fixation	620 (71.2%)	222 (68.3%)	398 (72.9%)	
Fixation	251 (28.8%)	103 (31.7%)	148 (27.1%)	
Size of hernia—no. (%)				0.456
≤ 1 finger	327 of 1006 (32.5)	136 of 325 (41.8)	191 of 546 (35.0)	
2 fingers	576 of 1006 (57.3)	194 of 325(60.0)	382 of 546 (70.0)	
≥ 3 fingers	103 of 1006 (10.2)	39 of 325 (12.0)	64 of 546 (11.7)	

Fisher’s exact test was used for categorical data, and the Kruskall-Wallis test was used for continuous data

### Recurrence

Of the 546 responders, 44 (8.1%, 95% CI: 6.0–11.0%) patients suspected a recurrence. Four (0.7%, 95% CI: 0.2–2%) patients underwent hernia repair for recurrence. Five patients refused the invitation to a follow-up visit, one was not contactable, and one did not wish to be contacted. One of the patients randomly selected for survey validation had signs that could not rule out a recurrence but refused the offer of clinical examination. Clinical or radiological findings confirmed recurrence in 9 of the 37 remaining patients who suspected a recurrence. Recurrence was thereby 13 out of 546 patients (2.4%; 95% CI: 1.4–4.1%). In the sensitivity analyses, i.e., worst-case scenario, recurrence was 21 out of 546 patients (3.8%, 95% CI: 2.5–5.8%).

We found no significant difference in recurrence stratified by fixation (Table 2). The negative and positive predictive values for self-reported recurrence were 1.00 (95% CI: 0.91–1.00) and 0.20 (95% CI: 0.10–0.35). The sensitivity analysis’ corresponding estimates were 0.97 (95% CI: 0.86–1.00) and 0.36 (95% CI: 0.22–0.52).

**Table 2:** Recurrence and reoperation rates

	All patients *N* = 546	No fixation *N* = 398	Fixation *N* = 148	*p* value
Recurrence rate	13 (2.4%)	10 (2.5%)	3 (2.0%)	1.00
Hernia repair for recurrence	4 (0.7%)	3 (0.8%)	1 (0.7%)	1.00
Recurrence in sensitivity analyses	21 (3.8%)	15 (3.8%)	6 (4.1%)	0.81

*p* value refers to comparing patients undergoing hernia repair with or without mesh fixation

The BMI of patients with recurrences was below 30, except for one patient. There were two L3 (lateral hernia, three fingers wide) and one M3 hernia (medial hernia, three fingers wide). The rest of the hernias were L1-2 and M1-2. Only one patient had an inguinoscrotal hernia.

### Pain

Forty (7.3%, 95% CI: 5.4–9.8%) patients reported pain at the time of the questionnaire. Three patients could not be contacted, four did not wish to be contacted, and one patient had undergone reoperation for recurrence. At the time of clinical follow-up (phone call), 17 (3.1%, 95% CI: 1.9–5.0%) patients still had pain, 4 female (5.5%) and 13 male (2.7%) patients. Their median age 61 years (36-86). Fourteen (2.6%, 95% CI: 1.4–4.3%) patients reported pain 1–4 times per month, which did not affect daily activity, and three patients (0.5%, 95% CI: 0.1–1.7%) experienced daily pain. They did not suspect a recurrence. One patient was treated with cryotherapy, which reduced the pain. One patient stopped playing sports, and one patient had to retire from work because of the intensity of the pain. Their median age was 61 years (45-75). The latter two reported preoperative pain similar to the postoperative pain. They were assessed by experts in chronic postoperative pain who concluded that the pain most likely was not correlated to the hernia repair.

There were no significant differences in chronic postoperative pain between fixation and no fixation of the mesh (Table 3).

**Table 3:** Postoperative pain reported by the patients in the survey

	All *N* = 546	No fixation *N* = 398	Fixation *N* = 148	*p* value
Groin pain > 3 months—no (%)	59 (10.8%)	46 (11.6%)	13 (8.8%)	0.438
Groin pain at the time of survey—no (%)	40 (7.3%)	33 (8.3%)	7 (4.7%)	0.195
Groin pain every day at the time of survey—no (%)	13 (2.4%)	12 (3.0%)	1 (0.7%)	0.259
Groin pain when sitting—no (%)	26 (4.8%)	22 (5.5%)	4 (2.7%)	0.346
Median groin pain when sitting (IQR)—NRS	5 (3–7)	5 (3–7)	5 (4–5)	0.693
Groin pain when moving—no (%)	30 (5.5%)	24 (6.0%)	6 (4.1%)	0.754
Median groin pain when moving (IQR)—NRS	4 (3–7)	5 (3–7)	4 (3–4)	0.529

*IQR* Interquartile range, *NRS* Numeric rating scale

### Hematoma or seroma

Out of 546 patients, postoperative hematoma was reported by 109 (20.0%, 95% CI: 16.8–23.5%) patients. Postoperative seroma was reported by 41 (7.5%, 95% CI: 5.6–10.0%) patients. There was no difference in self-reported postoperative hematoma (*p* = 0.470) or seroma (*p* = 0.279) between the fixation and non-fixation groups.

## Discussion

In this patient-reported follow-up study, we found low recurrence and postoperative pain rates compared to other studies [23, 24]. The recurrence rate was 2.4% and 3.8% in the sensitivity analysis. Pain affecting daily activity was 0.7%. Due to low discomfort from the recurrent hernia, only 33% had hernia repair for recurrence. This confirms that the reoperation rate is not synonymous with the recurrence rate.

A prospective study with patients undergoing inguinal hernia repair between 2009 and 2015 found that 11% of the patients underwent hernia repair for recurrence [25]. Only 38% of the recurrences occurred within 5 years after primary surgery, 57% within 10 years. They concluded that patients need decades of follow-up to assess “true” recurrence rates. Most studies have a follow-up time of 1–5 years, with recurrence rates varying from 0.5 to 15%. Our median follow-up time was 34 months. Further follow-up will be done to assess the 10-year recurrence rates.

A study from 2007 with 148 patients aimed to classify pain following open and laparoscopic hernia repair. The incidence of persistent pain was 10.8% after TAPP [26]. Another later study showed that 4.9% of the patients operated with a self-gripping mesh with the TAPP technique had mild discomfort after one year and 1% had moderate discomfort [3]. Findings that align with the results of our study. It is of great importance that surgeons identify the patients with a larger risk of chronic pain after hernia repair. Patients with severe daily pain should be examined for other pathology, and all patients must be thoroughly informed of the risk of chronic pain. Our department advises against hernia repair unless the hernia impairs activity.

Postoperative groin hematoma was reported by 20.0% of the patients with no association with mesh fixation. The high rate could not be clinically verified in our setup. The study design is a limitation as the patients might be unable to discriminate between hematomas and suggillations. As a standard, we administer antithrombotic medication Tinzaparin 3500 IE at the end of surgery. To our knowledge, no evidence supports postoperative thromboembolic prophylaxis after hernia repair in a day-surgical setting. A group of surgeons and anesthesiologists in our region are working together to recommend a new approach.

The patients reporting seroma (7.5%) are comparable to previously reported seroma incidences [27]. Currently, there are no evidence-based recommendations on how to avoid seroma after TAPP. None of our patients with hematoma or seroma needed treatment.

### Strengths and limitations

The National Person Register and each Danish resident’s unique civil registration number ensured that all patients who were alive and still resident in Denmark received the questionnaire via the secured email. The response rate of 62.7% is fairly high and comparable to similar studies after benign surgery conducted at NOH [28, 29]. Non-responding is one of the major biases when conducting a survey study. A US study showed that non-responders were predominantly found in the group of young age, male sex, and being a member of a minority group [30]. Our study had a nearly equal amount of male and female non-responders with a median age of 57 (IQR 44.00; 72.00) years. Since the questionnaire was sent out several months to years after the surgery, there is a risk of recall bias, mainly affecting the rate of early pain, hematoma, and seroma.

Follow-up after benign surgery is challenging. Many departments lack the resources for follow-up assessments on every patient, and patients often decline an invitation. Therefore, patient-reported data in surveys can contribute to the knowledge of postoperative outcomes after surgery. Self-administered surveys are applicable as follow-up on pain but are less applicable when assessing recurrence. The nearly fourfold self-reported overestimation of recurrence shows that some patients might confuse discomfort with recurrence. Clinical examination is therefore essential to assess the recurrence rate.

We found acceptable low recurrence and postoperative pain rates with no difference associated with mesh fixation. We found a tolerable self-reported incidence of seroma compared to other studies but a potentially biased high incidence of hematoma. With its self-adherent qualities, the Cousin mesh is safe and efficient to use without fixation.

## Data Availability

Personal data was handled according to the General Data Protection Regulation of the European Union, the Danish Personal Data Processing Act, and the Health Act. The database is reported to the Data Protection Agency of the Capital Region of Denmark.
